# Cellular responses to deproteinized bovine bone mineral biofunctionalized with bone-conditioned medium

**DOI:** 10.1007/s00784-020-03528-6

**Published:** 2020-09-01

**Authors:** Ludovica Parisi, Daniel Buser, Vivianne Chappuis, Maria B. Asparuhova

**Affiliations:** 1 Laboratory of Oral Cell Biology, Dental Research Center, School of Dental Medicine, University of Bern, Freiburgstrasse 3, 3010 Bern, Switzerland; 2 Department of Oral Surgery and Stomatology, School of Dental Medicine, University of Bern, Freiburgstrasse 7, 3010 Bern, Switzerland; 3 Centro Universitario di Odontoiatria, Dipartimento di Medicina e Chirurgia, University of Parma, Via Gramsci 14, 43126 Parma, Italy; 4 Present Address: Laboratory of Oral Molecular Biology, Dental Research Center, Department of Orthodontics and Dentofacial Orthopedics, University of Bern, Freiburgstrasse 3, 3010 Bern, Switzerland

**Keywords:** Bone augmentation, Autologous bone, Bone substitute, Osteogenesis, Growth factors, Gene expression

## Abstract

**Objectives:**

The aim of the study was to investigate whether the osteoinductive properties of bone-conditioned medium (BCM) harvested from cortical bone chips within a clinically relevant short-term period can enhance the biologic characteristics of deproteinized bovine bone mineral (DBBM) in vitro.

**Materials and methods:**

To assess the biofunctionalization of DBBM, the adhesive, proliferative, and differentiation properties of mesenchymal stromal ST2, pre-osteoblastic MC3T3-E1, and primary bone-derived cells grown on BCM-coated DBBM were examined by crystal violet staining of adherent cells, BrdU ELISA, and qRT-PCR, respectively.

**Results:**

BCM extracted within 20 min or 24 h in either Ringer’s solution (BCM-RS) or RS mixed with autologous serum (BCM-RS + S) increased the adhesive properties of all three cell types seeded on DBBM. The 20-min BCM-RS preparation appeared more potent than the 24-h preparation. BCM-RS made within 20 min or 24 h had strong pro-proliferative effects on all cell types grown on DBBM. RS + S alone exhibited a considerable pro-proliferative effect, suggesting an impact of the serum on cellular growth. DBBM coated with BCM-RS or BCM-RS + S, made within 20 min or 24 h each, caused a significant induction of osteogenic differentiation marker expression with a higher potency of the BCM-RS + S. Finally, a strong additive effect of fresh bone chips combined with BCM-coated DBBM on the osteogenic differentiation of the three cell types was observed.

**Conclusions:**

Altogether, the data strongly support the biofunctionalization of DBBM with BCM extracted within a clinically relevant time window of 20 min.

**Clinical relevance:**

Pre-activation of non-osteoinductive biomaterials with BCM, prepared from autologous bone chips during a guided bone regeneration (GBR) procedure, bears the potential of an optimal treatment modality for bone defects in daily practice.

**Electronic supplementary material:**

The online version of this article (10.1007/s00784-020-03528-6) contains supplementary material, which is available to authorized users.

## Introduction

Reconstruction of maxillofacial and oral bone defects might represent a clinical challenge that requires the use of bone substitutes [1]. The indications for using bone grafts range from minor fenestration defects to bridging major continuity defects in the facial skeleton. Despite the advances in the generation of new bone substitute materials, autografts are largely considered as the gold standard in osseous reconstructive surgery [2–4]. Indeed, bone derived from the patient own body is the only bone graft that possesses osteogenic, osteoinductive, and osteoconductive properties, thus providing viable osteogenic precursor cells, growth factors as well as physical scaffolding structure to foster new bone formation, and revascularization of the augmented volume [5]. Furthermore, recent laboratory-based evidence supports a paracrine function of the autogenous bone realized through a spectrum of secretory proteins [6] that possess the potential to target cellular processes in various cell types actively supporting the graft consolidation process in vivo [7–9].

Although autografts are considered superior for their consolidation, harvesting requires technical expertise and, depending on the need for an additional harvesting site, can be associated with increased surgical time, hematoma, postoperative pain, neurovascular injury, increased risk of infection, and esthetic deformity at the graft extraction site [10–12]. In addition, often intraoral sites do not provide sufficient quantities of bone for a grafting medium on large alveolar defects [11]. To overcome the shortcomings of the autograft alone, a guided bone regeneration (GBR) technique utilizing a combination of autologous bone chips with a bone graft substitute, such as deproteinized bovine bone mineral (DBBM), and a bioabsorbable collagen membrane was developed [13–15]. The two (autologous and xenogenic) bone fillers, a combination termed composite graft, are applied either in two layers or mixed. This bone augmentation technique has been successfully applied for a number of indications including early implant placement with simultaneous contour augmentation in the esthetic zone [3, 16–18], horizontal and vertical bone augmentation called sausage technique [19, 20], and for sinus floor elevation procedures in the posterior maxilla [21]. In addition, the GBR technique using a 2-layer composite graft has demonstrated good long-lasting stability of the peri-implant hard and soft tissues, and excellent esthetic outcomes [3, 18, 22].

The DBBM applied in the GBR procedure combines good osteoconductivity with a low substitution rate [23, 24]. In addition, the lack of organic matrix makes DBBM fully biocompatible, showing minimal risks for foreign body reactions in patients [25, 26]. However, the processing limits the biological activity of the DBBM, namely its osteogenic and osteoinductive properties. To address this limitation, the paracrine function of the autograft has been utilized in the GBR by pre-hydration of the DBBM and collagen membranes with bone-conditioned medium (BCM) [27–29]. BCM is a mixture of patient’s own blood and physiological solution enriched with autogenous growth factors [30]. These growth factors are released from autologous bone chips harvested from an intraoral site and stored in the mixture of autologous blood and saline for the time of the surgical site and implant bed preparation.

Recently, our laboratory has revealed part of the biological mechanisms that underlie the successful outcomes from GBR procedures utilizing BCM derived from autologous bone [6–9, 31–33]. It has been demonstrated that the technique by which the autologous bone chips are harvested may significantly influence the cellular viability and the release of growth factors [34]. BCM extracted from bone scraper or bone mill samples has revealed significantly higher expression of growth factors such as vascular endothelial growth factor (VEGF) and bone morphogenetic protein-2 (BMP-2) compared to samples prepared by piezosurgery or bone drilling [34, 35]. Moreover, higher amounts of newly formed bone in association with autografts harvested by bone mill compared to grafts harvested by bone scraper or piezosurgery were also demonstrated in a histomorphometric study in vivo using a minipig model [36].

More recently, we have shown that the time frame for the BCM extraction from cortical bone affects the BCM composition and the behavior of osteoprogenitors treated with it [33]. In particular, we have demonstrated a significant and very fast release of transforming growth factor-β1 (TGF-β1) from autogenous bone chips within 10 min versus a delayed BMP-2 release from 40 min on. BCMs harvested within short terms (10, 20, or 40 min), corresponding to the typical time of a surgical procedure, significantly enhanced the proliferative capacity and collagen matrix production of BCM-treated osteoprogenitors. BCMs extracted within longer periods (1, 3, or 6 days) exhibited a great capacity to induce the later stages of osteoblast differentiation and matrix mineralization due to the combined activity of TGF-β1 and BMP-2.

As a clinically relevant translation of these investigations, we hypothesized that short-term coating of DBBM with short-term extracted BCM potentiates the beneficial properties of the biomaterial. More specifically, the aim of the present study was to investigate whether the biological activity of the BCM can be transferred onto the DBBM in vitro, leading to induced osteogenic properties of mesenchymal stromal cells, pre-osteoblastic cells, and primary human bone-derived cells. The study further aimed to investigate if a synergy between BCM-coated DBBM and freshly harvested bone chips could be detected, thus providing justification for a clinical approach combining autologous bone, BCM, and DBBM, which would completely utilize the osteoinductive properties of the autograft.

## Materials and methods

### BCM preparation and DBBM coating

**Fig. 1:**
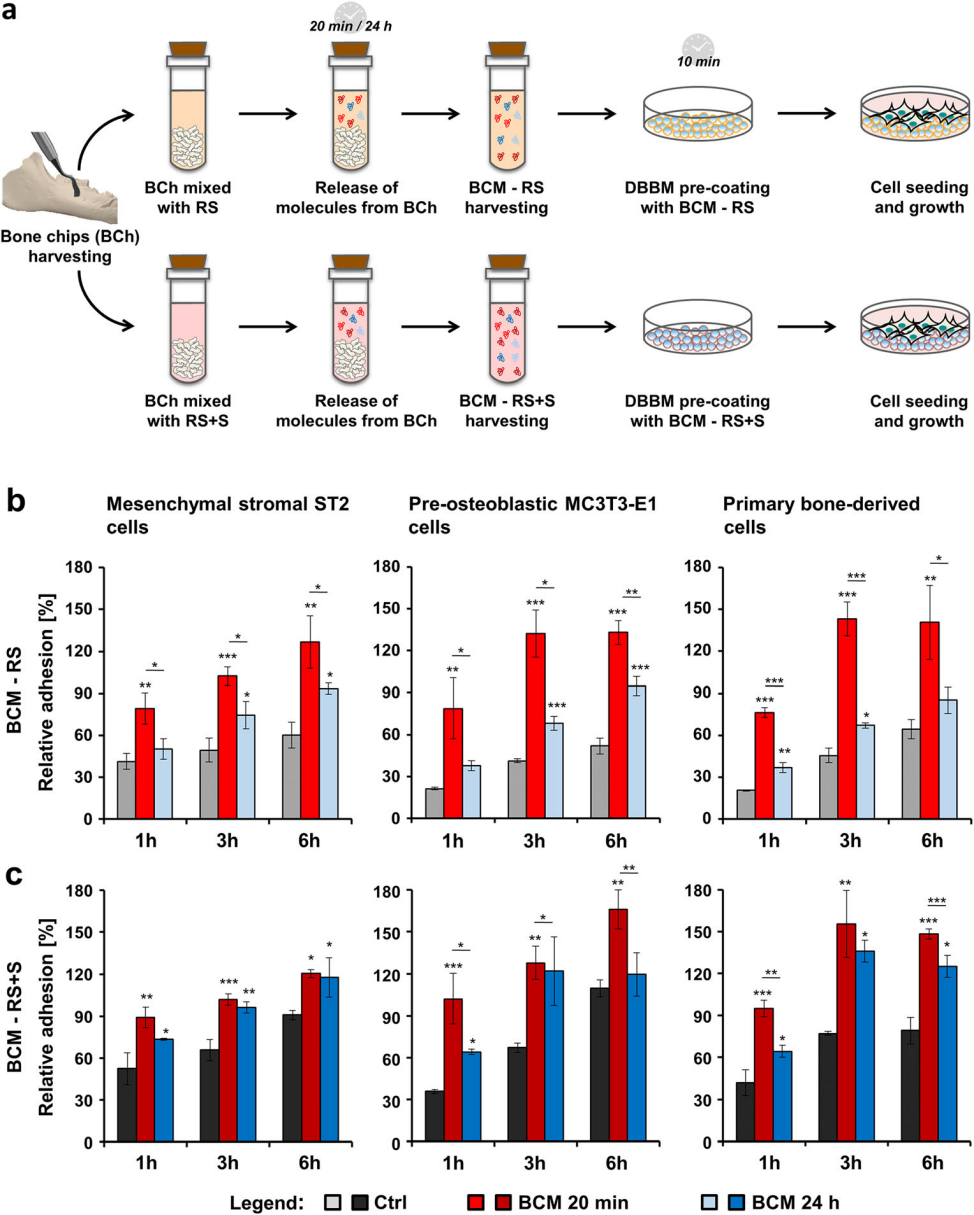
Increased adhesive properties of bone-related cell cultures grown on BCM-coated DBBM. **a** Schematic representation of the experimental set-up utilized throughout the study. The following steps are illustrated: (1) harvesting of cortical bone chips (BCh) from the buccal side of fresh pig mandibles using a bone scraper; (2) mixing of the BCh with extracting solutions consisting of Ringer’s solution (RS) or a 1:1 mixture of Ringer’s solution and autologous serum (RS + S); (3) incubation of the resulting mixtures for 20 min or 24 h during which time release of molecules from the BCh occurs; (4) harvesting of two types of bone-conditioned medium (BCM) labeled as BCM-RS and BCM-RS + S; (5) coating of deproteinized bovine bone mineral (DBBM) with the respective BCM preparations for 10 min prior to (6) cell seeding and growth. **b**, **c** Adhesion rate of ST2, MC3T3-E1, and primary bone–derived cells plated on DBBM coated with BCM-RS (b) or BCM-RS + S (c) prepared within 20 min or 24 h each. Controls (Ctrl) represent cells of each cell type seeded on BCM-free DBBM hydrated with RS or RS + S. Adhesion rates were assessed by crystal violet assay at 1, 3, and 6 h. Experimental values were normalized to the values obtained for the total number of seeded control cells, taken as 100% adhesion. Means ± SD from three independent experiments and significant differences to control cells at each time point unless otherwise indicated, ***P < 0.001, **P < 0.01, *P < 0.05 are shown

The preparation of BCM was described previously [30]. In brief, cortical bone chips from the buccal side of fresh pig mandibles (Slaughterhouse: Küng Metzgerei, Toffen, Switzerland) were harvested by using a bone scraper (Hu-Friedy, Rotterdam, the Netherlands) and soaked in extracting solutions for 20 min or 24 h (Fig. 1a). Extracting solutions, consisting of either Ringer’s solution (RS) or RS mixed with autologous serum (RS + S) in a 1:1 ratio, were supplemented with 1% antibiotics/antimycotics (AA; ThermoFisher Scientific, Basel, Switzerland). A ratio of 1 g bone chips per 2 ml medium (50% weight/volume) was utilized. Two types of media, abbreviated BCM-RS and BCM-RS + S, were collected from three independent preparations. The BCMs were sterile filtered and kept frozen at − 80 °C until experimental cell seeding.

DBBM (Geistlich Bio-Oss®) granules in the 0.25–1-mm range size were kindly provided by Geistlich Pharma (Wolhusen, Switzerland). Prior to cell seeding, 35 or 200 mg of DBBM granules (resulting in a layer of a 3-mm height) were placed on the bottom of 96- or 24-well ultra-low attachment plates (Corning, NY, USA), respectively, and coated for 10 min with each of the BCM preparations. Cells seeded on BCM-free DBBM, pre-hydrated with RS or RS + S, were used as controls (Ctrl) throughout the study. Corning® ultra-low attachment plates were our product of choice among three different brands tested because it ensured 0% cell attachment on the plastic surface even after coating with BCM.

A 3D-culturing system was also adopted in the present study. DBBM granules in the 24-well culture system were prepared as described above, and coated with either RS + S or the 20-min BCM-RS + S preparation. Immediately after cell seeding, a Nunc™ polycarbonate cell culture insert (ThermoFisher Scientific) with a 0.4-μm pore size was placed in the cell culturing well and filled with 0.5 g of fresh bone chips as depicted in Fig. 7a.

### Cell culture

Mesenchymal stromal ST2 cells derived from mouse bone marrow were obtained from RIKEN Cell Bank (Tsukuba, Japan). Pre-osteoblastic MC3T3-E1 cells derived from a C57BL/6 mouse calvaria were obtained from ECACC collection (Sigma-Aldrich, Buchs, Switzerland). Both cell lines were grown in Dulbecco’s modified Eagle medium (DMEM) supplemented with 10% fetal calf serum (FCS; Invitrogen, Zug, Switzerland) and 1% AA (ThermoFisher Scientific). Cells were starved in 0.3% FCS/DMEM for 24 h before culturing on DBBM.

Primary human bone–derived cells were obtained from three donors using a modification of previously described techniques [37]. Fresh bone particles harvested from the retromolar area using a bone scraper were retrieved from anonymous and systemically healthy individuals, who had undergone implant placement with GBR procedure at the Department of Oral Surgery and Stomatology, following signed informed consent and approval by the Ethics Committee, Bern, Switzerland. In brief, after extensive washing in excess of sterile PBS for complete removal of blood and dislodged cells, the bone chips were digested using 0.2% collagenase type II (Worthington Biochemical Corporation, Lakewood, NJ, USA) in Hanks’ balanced salt solution for 30 min, in a humidified atmosphere of 5% CO_2_ at 37 °C. Complete culturing medium was used to stop the enzymatic reaction and specimens were placed into a gentleMACS™ C Tube (Miltenyi Biotec, Solothurn, Switzerland). GentleMACS Dissociator (Miltenyi Biotec) was used for automated dissociation of the specimens to obtain a single-cell suspension. The suspension was subsequently passed through Falcon® 70 μm Cell Strainer (Corning) before culturing in complete minimum essential medium Eagle–alpha modification (α-MEM; Thermo Fischer Scientific) supplemented with 10% FCS and 1% AA. Cells that had not undergone more than five passages were starved in 0.3% FCS/α-MEM for 24 h before culturing on DBBM. Data in each data set were obtained from three independent experiments performed with primary bone–derived cells from three different cell donors. Characterization of the primary human bone–derived cultures, according to published classifications [38, 39] and in comparison with the ST2 and MC3T3-E1 strains, revealed them as a mixture of cells with osteoblastic and preosteocytic phenotype (Electronic Supplementary Material, Figure S1). However, no expression of sclerostin, which is considered as a marker of mature osteocytes, was detected in any of the three cell types.

For differentiation experiments followed by RNA analyses, complete media were supplemented with 50 μg/mL ascorbic acid (Invitrogen, Zug, Switzerland) and 2 mM β-glycerophosphate (Invitrogen) as it was described previously [33].

### Cell adhesion assay

The adhesion capacity of ST2, MC3T3-E1, and primary bone–derived cells cultured on BCM-coated DBBM was determined by crystal violet (Sigma-Aldrich) staining of adherent cells. In brief, 5 × 10^4^ cells/well were plated in duplicate, in complete medium on DBBM in ultra-low attachment 96-well plates and allowed to adhere for 1, 3, and 6 h. To test for a potential background staining produced by the BCM-coated DBBM, no cell controls were included in the assay (Electronic Supplementary Material, Figure S2a). After removal of culture medium, cells were extensively washed 3 times in PBS in order to remove any non-adherent cells, then fixed in 4% paraformaldhyde (PFA; Sigma-Aldrich) for 20 min at room temperature (RT), and stained with crystal violet solution (0.1%; Sigma-Aldrich) in PBS for 30 min at RT. After 8 washing cycles in deionized H_2_O to remove crystal violet excess, the dye bound to adherent cells was solubilized using a 10% (volume/volume) acetic acid (Sigma-Aldrich) on an orbital shaker at 150 rpm for 5 min at RT. The absorbance of the eluate was measured at 570 nm using an EL808 reader (BioTek Instruments GmbH, Lucerne, Switzerland). To obtain a value for the total number of seeded cells as well as to correct for the background signal produced by the BCM, cells of each experimental group were processed in parallel in the following way: After removal of the culture medium, cells were directly fixed in 4% PFA omitting the 3 cycles of extensive wash in PBS. The rest of the procedure was performed as described above. Please, refer to the Electronic Supplementary Material, Figure S2b for more details on the quantitative assessment of the background staining produced by the BCM. Experimental values corrected for the background signal were normalized to the values obtained for the total number of seeded control cells, which were allowed to settle down for 1 h and processed as described above (conditionally taken as 100% adhesion). Data represent means ± SD from three independent experiments performed with each of the three cell types, in duplicates.

### Cell proliferation assay

Proliferation rates of cultured on BCM-coated DBBM ST2, MC3T3-E1, and primary bone–derived cells were determined using a 5-bromo-20-deoxyuridine (BrdU) incorporation ELISA (Roche, Basel, Switzerland) as described [40]. In brief, after starvation of 24 h, cells were plated in triplicate at 1 × 10^3^ cells/well, in 3% FCS-containing medium on DBBM in black ultra-low attachment 96-well plates and allowed to proliferate for 0, 24, 48, 72, and 96 h before a 2-h labeling with BrdU. Incorporation of BrdU into newly synthesized DNA was determined according to the manufacturer’s protocol using an Infinite® 200 luminometer (Tecan, Männedorf, Switzerland). Experimental values were normalized to the values of control cells at the time point 0. Data represent means ± SD from three independent experiments performed with each of the three cell types, in triplicates.

### RNA analysis by quantitative reverse transcription-polymerase chain reaction

For RNA analyses, cells were plated at 2.5 × 10^5^ cells/well, in differentiation medium on DBBM in ultra-low attachment 24-well plates. Total RNA from cells grown on BCM-coated DBBM in the absence or in the presence of fresh bone chips (Fig. 7a) for 1, 3, and 7 days was isolated using Trizol (Thermo Fisher Scientific) according to the manufacturer’s protocol. The extracted RNA was additionally purified by using the RNeasy MinElute Cleanup Kit (Qiagen, Basel, Switzerland). RNA, quantified on a NanoDrop 2000c instrument (ThermoFisher Scientific), was reverse transcribed and relative transcripts for collagen type I (Col1a1), runt-related transcription factor 2 (Runx2), alkaline phosphatase (Alpl), and osteocalcin (or bone gamma-carboxyglutamate protein 2, Bglap2) genes, normalized to Gapdh, were measured using FastStart Universal SYBR Green Master ROX (Roche), and the primer sequences listed in Electronic Supplementary Material, Tables S1 and S2. The two tables include four additional osteogenic marker genes that were used in the characterization of the primary human bone–derived cells (for more details, please refer to the Electronic Supplementary Material, Figure S1). For simplicity, the same non-capitalized symbols were used for both mouse and human genes throughout the paper except in Table S2, where human gene symbols were capitalized. qPCR was carried out in a QuantStudio 3 instrument (Applied Biosystems, Rotkreuz, Switzerland) using a standard thermal cycling profile. The efficiency ∆∆Ct method [41] was used to calculate gene expression levels normalized to Gapdh values and calibrated to values of controls at day 1. All samples were run in duplicates. Data represent means ± SD from three independent experiments performed with each of the three cell types.

### Statistical analysis

All grouped data are means ± SD. Statistical analysis was performed using GraphPad InStat Software (GraphPad, La Jolla, CA, USA), version 3.05. Multiple comparisons were completed using one-way analysis of variance (ANOVA) with Tukey’s post hoc test. Values of *P* < 0.05 were considered significant.

## Results

### Increased adhesive properties of bone-related cell cultures grown on BCM-coated DBBM

Figure 1a depicts the experimental set-up used throughout the study. Bone chips harvested from the buccal side of fresh pig mandibles were mixed with either RS or RS + S. The resulting BCMs, BCM-RS and BCM-RS + S, extracted for either 20 min or 24 h each, were used for a short 10 min-coating of DBBM prior to cell seeding and growth for different time intervals.

We first investigated the adhesive properties of the mesenchymal stromal ST2, pre-osteoblastic MC3T3-E1, or primary human bone–derived cells seeded either on control BCM-free DBBM hydrated with RS or RS + S, DBBM coated with the 20-min BCM preparation, or DBBM coated with the 24-h BCM preparation. The BCMs were made in each of the two diluents, RS (Fig. 1b) or RS + S (Fig. 1c). Adhesion was followed over 1, 3, and 6 h using crystal violet assay. The results showed that in comparison to controls, BCM extracted within 20 min in RS significantly (*P* < 0.01) increased the adhesive properties of all three cell types seeded on DBBM (Fig. 1b). With few exceptions, the 24-h BCM-RS preparation also caused significant (*P* < 0.05) increase in the adhesive potential of the three cell types seeded on DBBM. Interestingly, in all three cell types, the 20-min BCM preparation appeared to be more potent than the 24-h preparation (compare red with blue bars, *P* < 0.05).

A similar behavior was detected for cells seeded on DBBM coated with BCM-RS + S prepared for either 20 min or 24 h (Fig. 1c). However, the 20-min BCM preparation appeared to be significantly more potent than the 24-h preparation in the case of MC3T3-E1 pre-osteoblasts (*P* < 0.05) at each time point and in the case of the primary bone–derived cells (*P* < 0.01) at 1 and 6 h. Interestingly, control cells seeded on BCM-free DBBM pre-coated with RS + S (dark gray bars, Fig. 1c) were characterized with higher but not significant relative adhesion values compared to control cells seeded on DBBM pre-hydrated with RS (light gray bars, Fig. 1b). This suggests that to a certain extent, serum proteins along with BCM contribute to the increased adhesive properties of the investigated cell types.

### Pro-proliferative properties of bone-related cell cultures grown on DBBM coated with BCM-RS

**Fig. 2:**
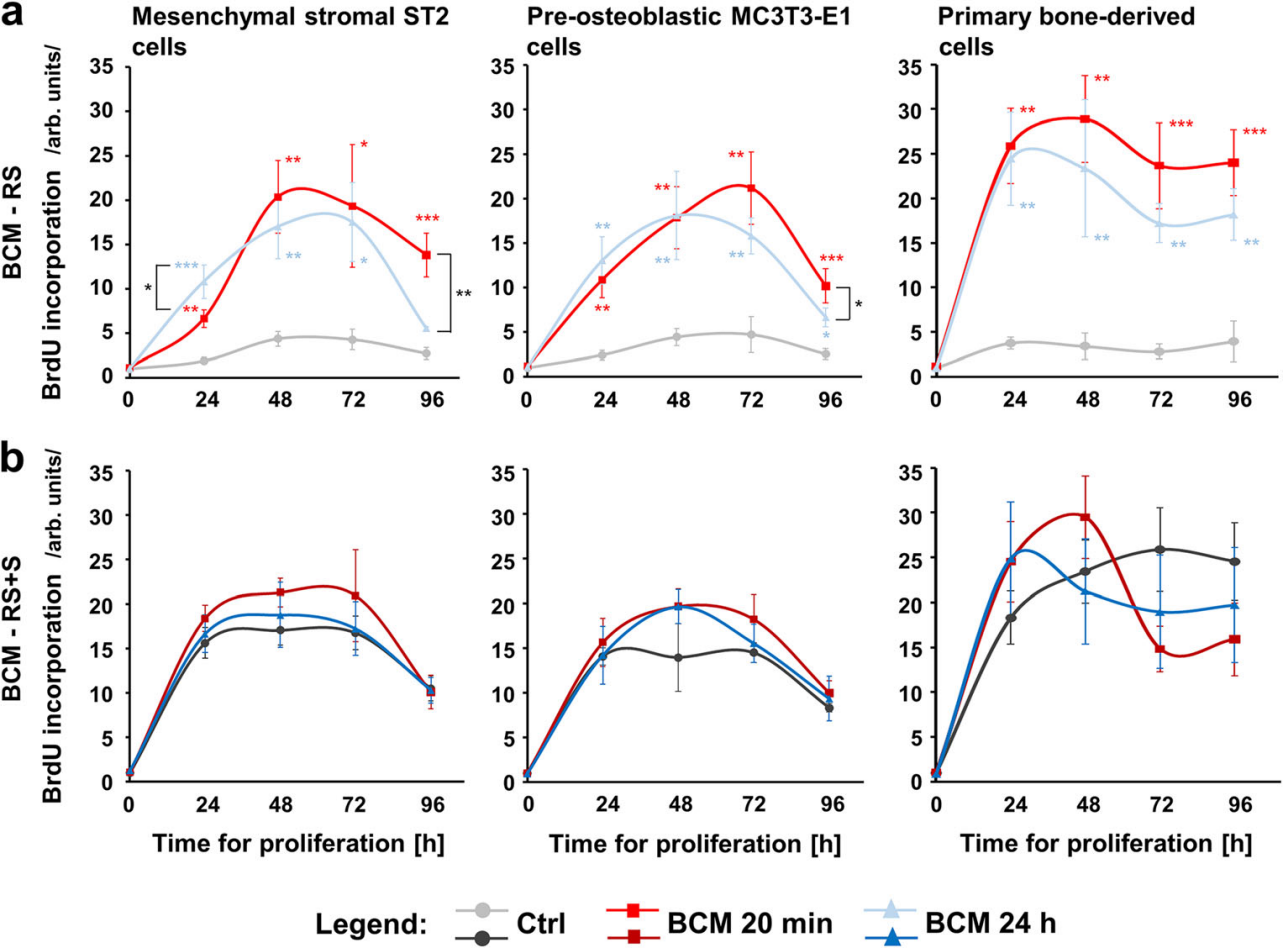
Proliferative properties of bone-related cell cultures grown on BCM-coated DBBM. **a**, **b** Proliferative ability of ST2, MC3T3-E1, and primary bone–derived cells grown on DBBM coated with BCM-RS (a) or BCM-RS + S (b), was assessed by BrdU incorporation into newly synthesized DNA immediately after plating (0 h) as well as at 24, 48, 72, and 96 h. Cells of each cell type grown on BCM-free DBBM that is hydrated with RS (a) or RS + S (b) are used as controls (Ctrl). Experimental values were normalized to the values of control cells at the time point 0. Means ± SD from three independent experiments and significant differences to control cells unless otherwise indicated, ***P < 0.001, **P < 0.01, *P < 0.05 are shown

We further evaluated the proliferative rate of ST2, MC3T3-E1, and primary bone–derived cells grown on BCM-coated DBBM (Fig. 2). Compared to control cells grown on DBBM hydrated with RS, cells grown on DBBM coated with BCM-RS preparations made within 20 min or 24 h showed a strong increase in BrdU uptake into newly synthesized DNA until a confluence was reached (Fig. 2a). Compared to the respective controls, the detected increase was in the range of 3.9–4.5-fold for ST2 and MC3T3-E1 cell lines and 6.0–7.5-fold for the primary bone–derived cultures grown on the BCM-coated DBBM. Most importantly, the short-term 20-min BCM preparation was as potent as the 24-h preparation except for ST2 cells after 24 h of growth, at which time point the 24-h BCM preparation exhibited a significantly higher (*P* < 0.05) proliferative rate than the 20-min preparation.

In contrast to the effects caused by the BCM-RS-coated DBBM, no significant differences in the proliferative rates of control cells grown on DBBM hydrated with RS + S and cells grown on DBBM coated with BCM-RS + S preparations made within 20 min or 24 h were detected for any of the three cell types over 5 days (Fig. 2b). Notably, for each of the three cell types, the growth rates of control cells seeded on RS + S-coated DBBM were considerably higher (in the range of 3.1–6.9-fold) compared to control cells seeded on RS-hydrated DBBM (Fig. 2a and b). This finding suggests a significant impact of the serum on the proliferative ability of the three cell types, which may be further extrapolated to the patient’s own blood used to store the autologous bone chips during the surgical tooth bed preparation.

### BCM-coated DBBM induces osteogenic differentiation of bone-related cell cultures

**Fig. 3:**
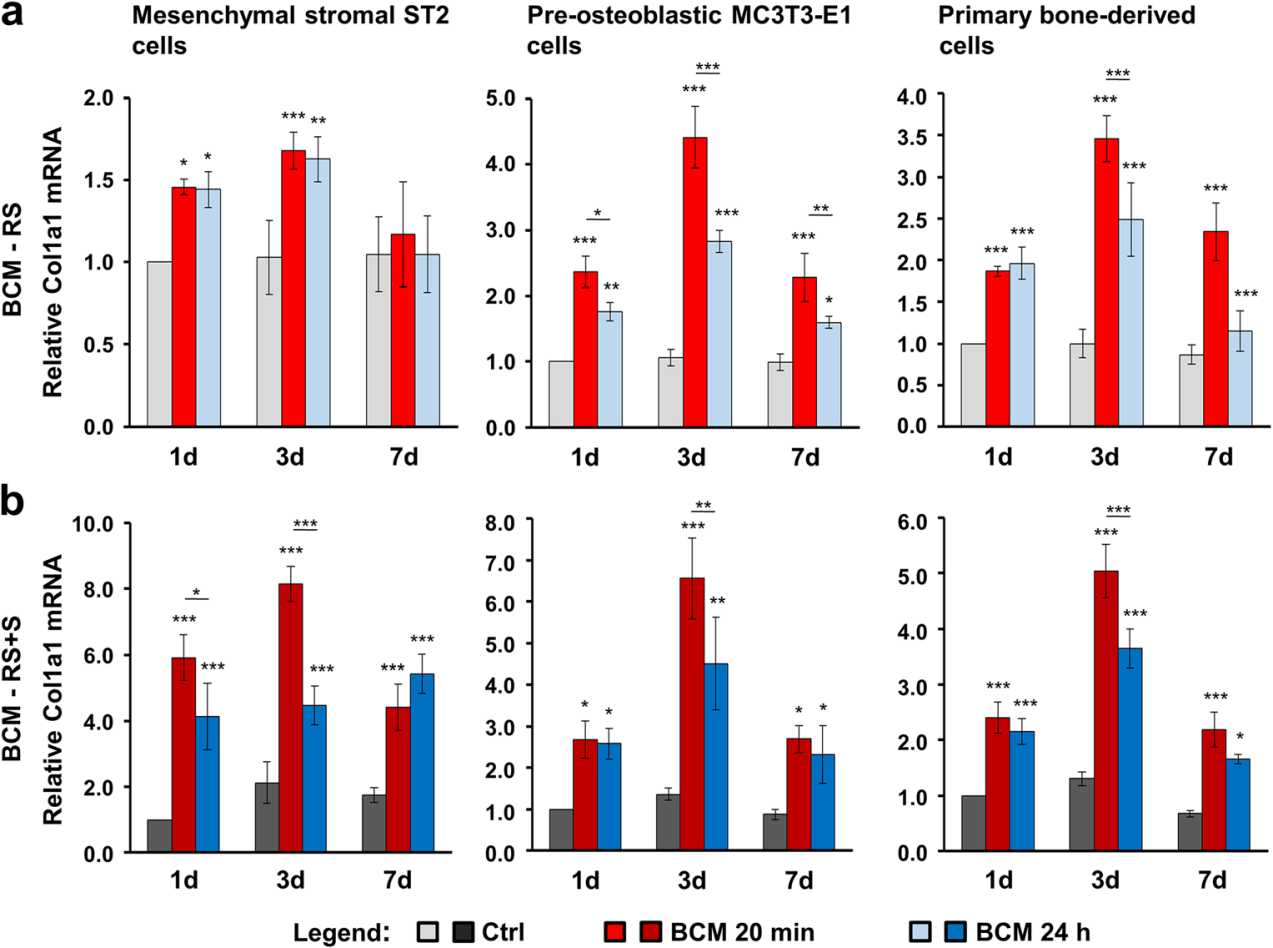
BCM-coated DBBM induces Col1a1 gene expression in bone-related cell cultures. **a**, **b** ST2, MC3T3-E1, and primary bone–derived cells were grown on DBBM coated with BCM-RS (a) or BCM-RS + S (b) for 1, 3, and 7 days before total RNA was isolated and analyzed for Col1a1 expression by qRT-PCR. Controls (Ctrl) represent cells of each cell type seeded on BCM-free DBBM hydrated with RS (a) or RS + S (b). Values normalized to Gapdh are expressed relative to the values of control cells at the “1 day” time point. Please note the differences in the scales of the *y*-axis. Data represent means ± SD from three independent experiments. Significant differences to the respective controls at day 1 unless otherwise indicated, ***P < 0.001, **P < 0.01, *P < 0.05

**Fig. 4:**
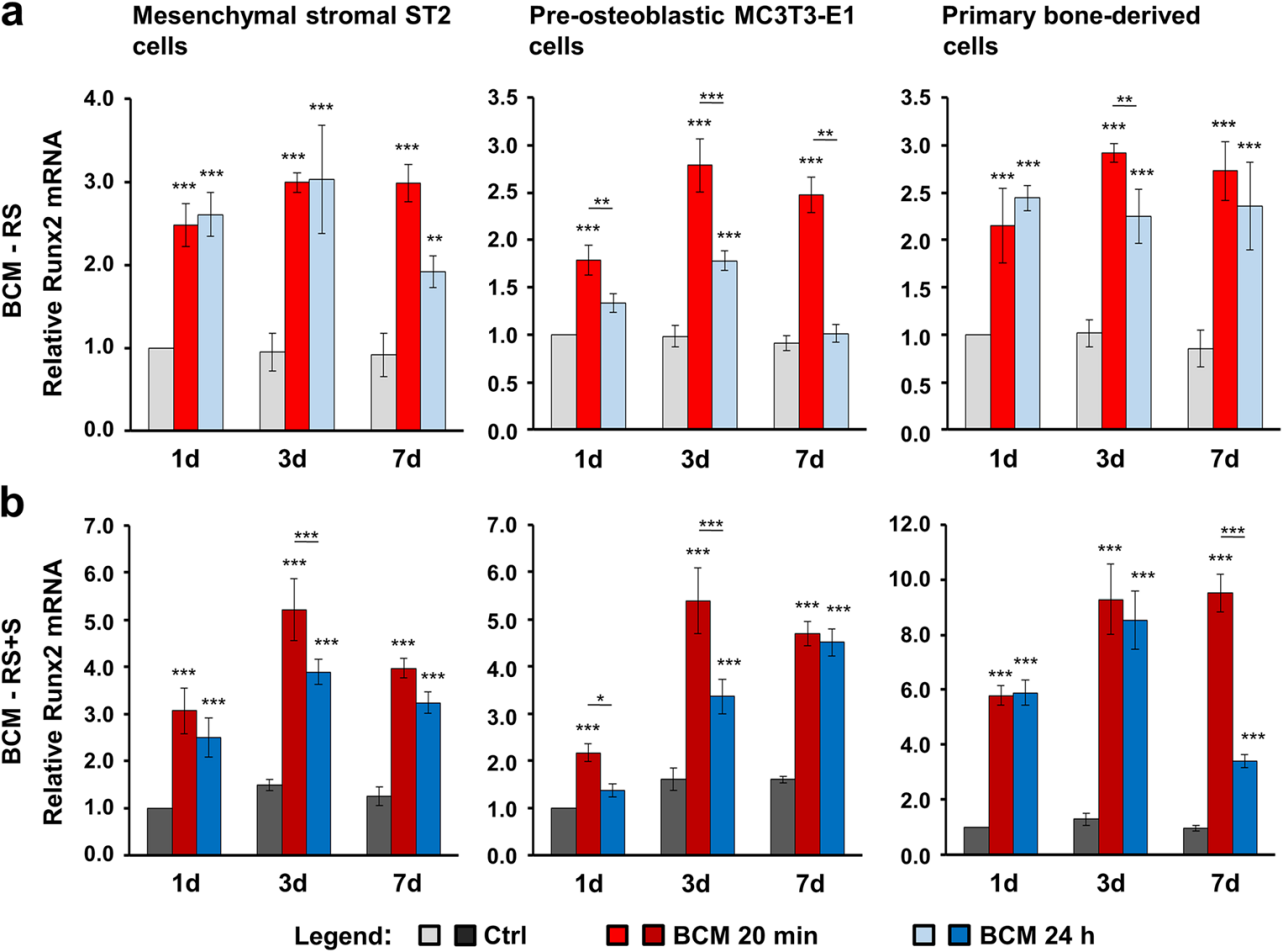
BCM-coated DBBM induces Runx2 gene expression in bone-related cell cultures. **a**, **b** ST2, MC3T3-E1, and primary bone–derived cells were grown on DBBM coated with BCM-RS (a) or BCM-RS + S (b) for 1, 3, and 7 days before total RNA was isolated and analyzed for the expression of the transcription factor gene Runx2 by qRT-PCR. Controls (Ctrl) represent cells of each cell type seeded on BCM-free DBBM hydrated with RS (a) or RS + S (b). Values normalized to Gapdh are expressed relative to the values of control cells at the “1 day” time point. Please note the differences in the scales of the *y*-axis. Data represent means ± SD from three independent experiments. Significant differences to the respective controls at day 1 unless otherwise indicated, ***P < 0.001, **P < 0.01, *P < 0.05

**Fig. 5:**
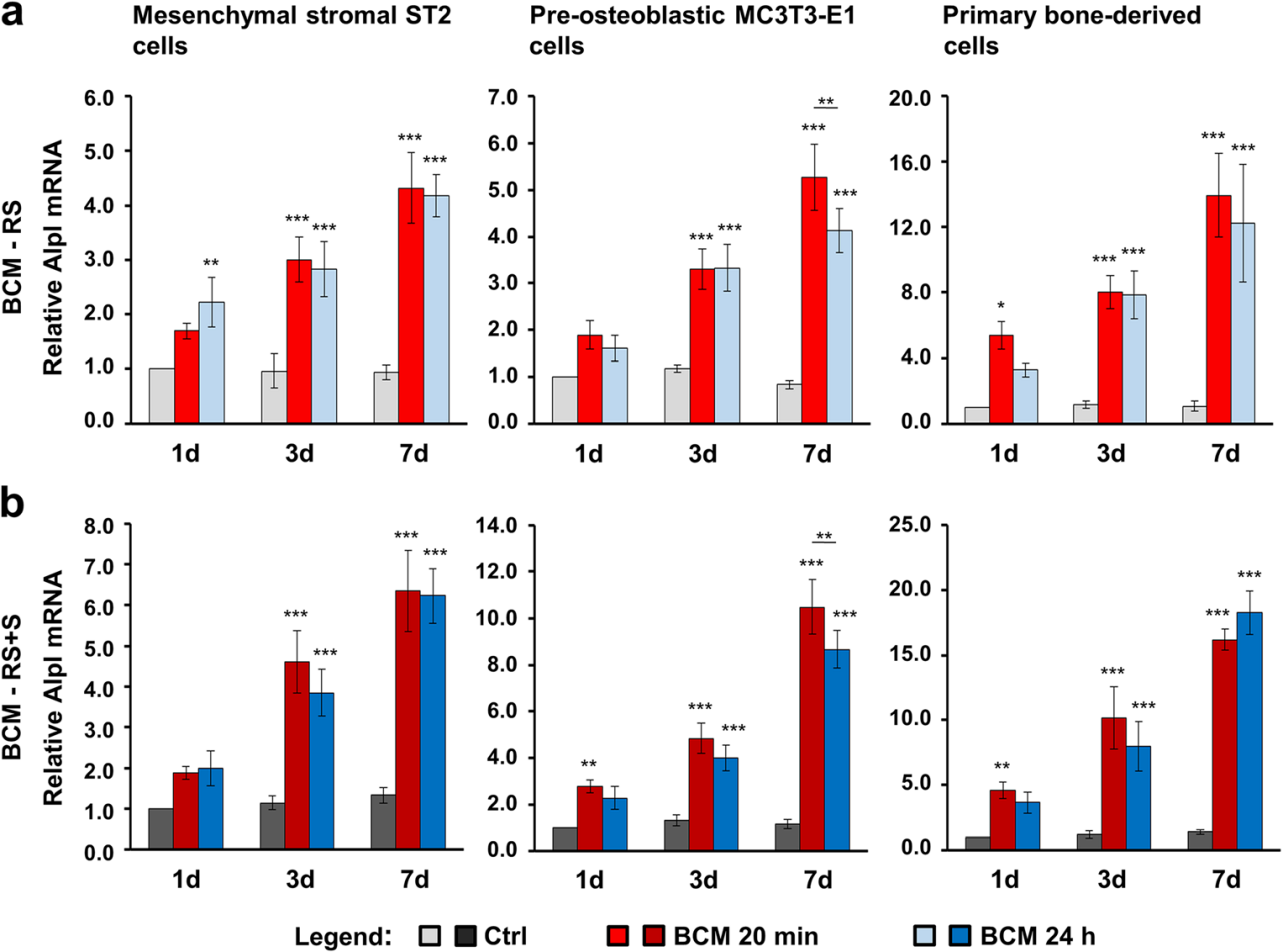
BCM-coated DBBM induces Alpl gene expression in bone-related cell cultures. **a**, **b** ST2, MC3T3-E1, and primary bone-derived cells were grown on DBBM coated with BCM-RS (a) or BCM-RS + S (b) for 1, 3, and 7 days before total RNA was isolated and analyzed for the expression of the osteogenic marker gene Alpl by qRT-PCR. Controls (Ctrl) represent cells of each cell type seeded on BCM-free DBBM hydrated with RS (a) or RS + S (b). Values normalized to Gapdh are expressed relative to the values of control cells at the “1 day” time point. Please note the differences in the scales of the *y*-axis. Data represent means ± SD from three independent experiments. Significant differences to the respective controls at day 1 unless otherwise indicated, ***P < 0.001, **P < 0.01, *P < 0.05

**Fig. 6:**
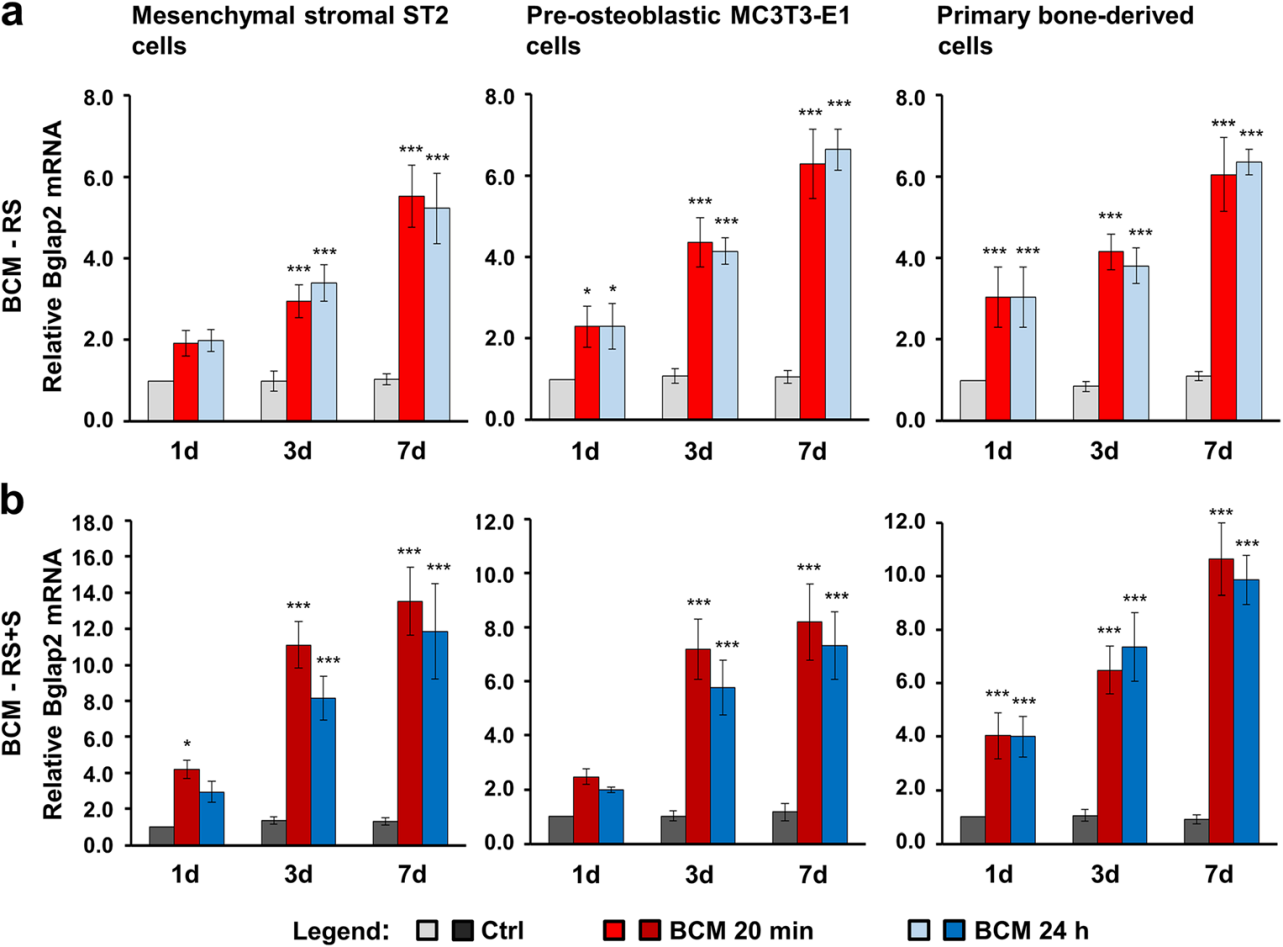
BCM-coated DBBM induces Bglap2 gene expression in bone-related cell cultures. **a**, **b** ST2, MC3T3-E1, and primary bone–derived cells were grown on DBBM coated with BCM-RS (a) or BCM-RS + S (b) for 1, 3, and 7 days before total RNA was extracted and analyzed for the expression of the osteogenic marker gene Bglap2 by qRT-PCR. Controls (Ctrl) represent cells of each cell type seeded on BCM-free DBBM hydrated with RS (a) or RS + S (b). Values normalized to Gapdh are expressed relative to the values of control cells at the time point “1 day.” Please note the differences in the scales of the *y*-axis. Data represent means ± SD from three independent experiments. Significant differences to the respective controls at day 1 unless otherwise indicated, ***P < 0.001, **P < 0.01, *P < 0.05

As a next step, we investigated the expression of osteogenic differentiation markers such as Col1a1 (Fig. 3), Runx2 (Fig. 4), Alpl (Fig. 5), and Bglap2 (Fig. 6) in cells grown on BCM-coated DBBM for 1, 3, and 7 days. DBBM coated with BCM made within 20 min or 24 h, in each of the two solutions (RS or RS + S), caused a significant increase in Col1a1 (Fig. 3) and Runx2 (Fig. 4) mRNAs above the levels expressed and detected in control cells grown on DBBM pre-hydrated with the diluents alone. The qRT-PCR analyses showed two clear trends. First, a tendency of a higher potency exhibited by the 20 min compared to the 24-h BCM preparation, especially in MC3T3-E1 and primary bone–derived cells (Figs. 3 and 4) as well as for individual time points in the ST2 cells grown on BCM-RS + S–coated DBBM (Fig. 3b and 4b), was observed. Second, a consistent fashion of a higher potency exhibited by the DBBM coated with BCM-RS + S in all three cell types was observed (refer to the difference in the scales between a and b in Figs. 3 and 4). Following a significant increase at days 1 and 3, Col1a1 expression at day 7 marked a decrease to the expression levels detected on day 1. In contrast, the expression of Runx2 generally reached a plateau at day 3, and thus remained high at day 7 (Fig. 4).

Compared to Col1a1 and Runx2, Alpl (Fig. 5) and Bglap2 (Fig. 6) genes showed a slightly different pattern of upregulated expression in cells grown on BCM-coated DBBM compared to control cells, with a clear trend of continuous upregulation over 7 days. However, with a small exception for Alpl expression in MC3T3-E1 cells at day 7, we detected no significant differences in the potential of the two BCM preparations (20 min vs. 24 h) to upregulate the mRNA levels of the two osteogenic differentiation markers (Figs. 5 and 6). The trend of a higher potency exhibited by the DBBM coated with BCM-RS + S preparations remained (refer to the difference in the scales between a and b in Figs. 5 and 6).

Altogether, the results suggest that DBBM adsorbs and accumulates factors contained in the BCM that influence both early and late stages of the differentiation process. The process is governed by the upregulated transcription factor Runx2, which characterizes osteoprogenitors and orchestrates the next steps of the osteogenic process, accompanied by the increased expression of Col1a1 and Alpl characterizing pre-osteoblasts, and finally by the upregulated osteoblast marker Bglap2.

### Additive effect of fresh bone chips and BCM-coated DBBM on the osteogenic differentiation of bone-related cell cultures

**Fig. 7:**
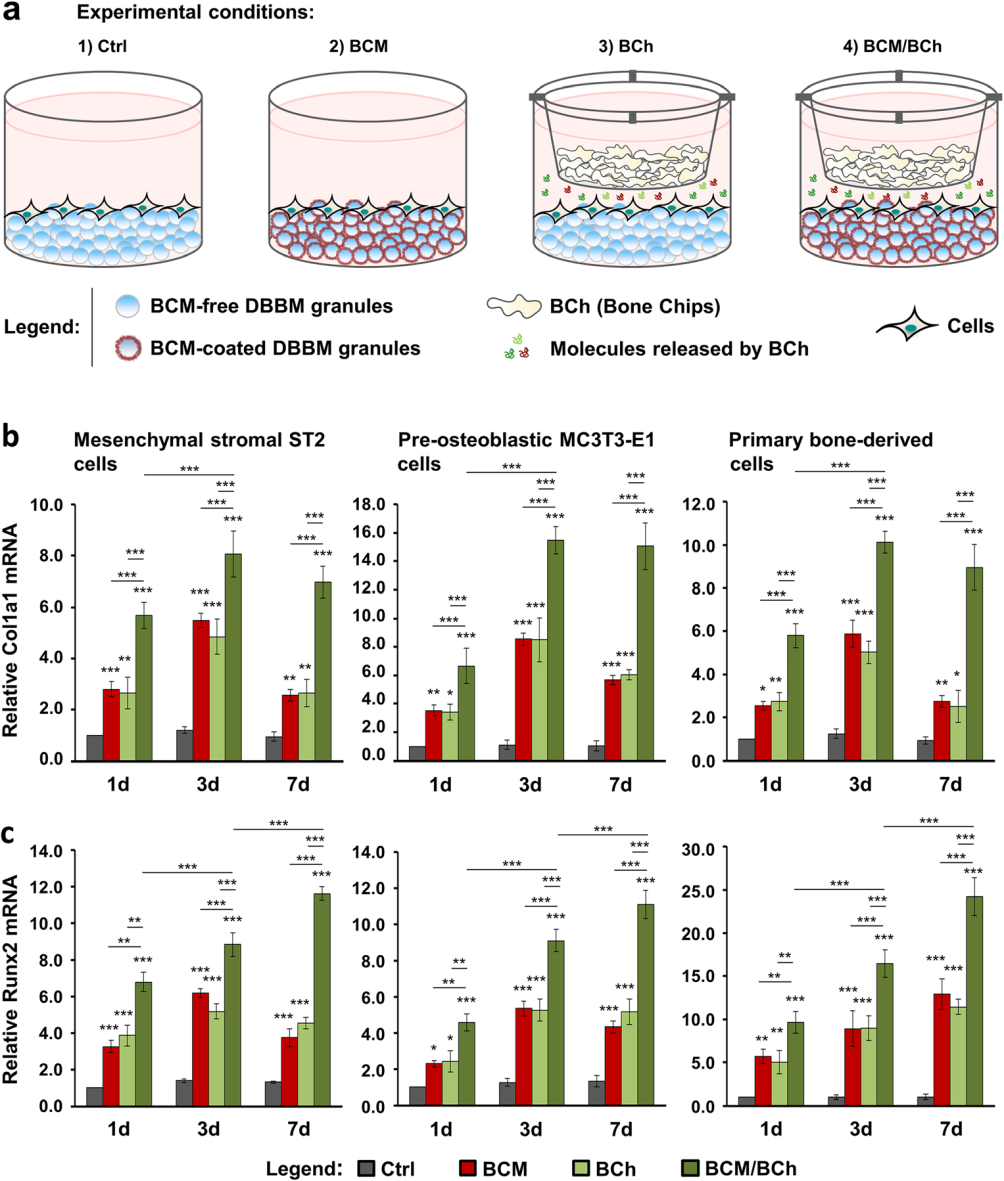
Additive effect of fresh bone chips and BCM-coated DBBM on the Col1a1 and Runx2 gene expression in bone-related cell cultures. **a** Schematic representation of the four experimental conditions, which are compared in (b). ST2, MC3T3-E1, and primary bone–derived cells are grown for 1, 3, and 7 days either on (1) BCM-free DBBM granules hydrated with RS + S, a condition labeled as control (Ctrl) or (2) DBBM granules coated with BCM prepared within 20 min in RS + S, a condition labeled as BCM. Experimental conditions (3) bone chips (BCh) and (4) BCM/BCh duplicate conditions (1) and (2) in the presence of fresh bone chips placed in a cell culture insert with a 0.4-μm pore size. **b**, **c** Effect of BCM-coated DBBM in the absence or presence of fresh bone chips on Col1a1 (b) and Runx2 (c) mRNA expression levels in ST2, MC3T3-E1, and primary bone–derived cells. Cells were grown under each of the four experimental conditions described in (a) for 1, 3, and 7 days before total RNA was isolated and analyzed by qRT-PCR. Values normalized to Gapdh are expressed relative to the values of control cells at the “1 day” time point. Please note the differences in the scales of the *y*-axis. Data represent means ± SD from three independent experiments. Significant differences to the respective controls at day 1 unless otherwise indicated, ***P < 0.001, **P < 0.01, *P < 0.05

Often, the GBR technique utilizes a combination of autologous bone, DBBM, and a collagen barrier membrane [3, 16–18, 21, 42]. To create an in vitro set-up that mimics as close as possible the clinical situation, we compared the differentiation potential of ST2, MC3T3-E1, and primary bone–derived cells grown under four different conditions: (1) control (Ctrl) condition, consisting of cells grown on BCM-free DBBM pre-coated with RS + S; (2) BCM condition, consisting of cells grown on DBBM coated with the 20-min BCM-RS + S preparation; (3) bone chips (BCh) condition, consisting of cells grown as in (1) but in the presence of fresh bone chips; and (4) BCM/BCh condition, consisting of cells grown in the combined presence of BCM-coated DBBM and fresh bone chips (Fig. 7a). The fresh bone chips in conditions (3) and (4) were placed in a cell culture insert with a 0.4-μm pore size, which allows transport of molecules released by the bone chips but no cell migration.

**Fig. 8:**
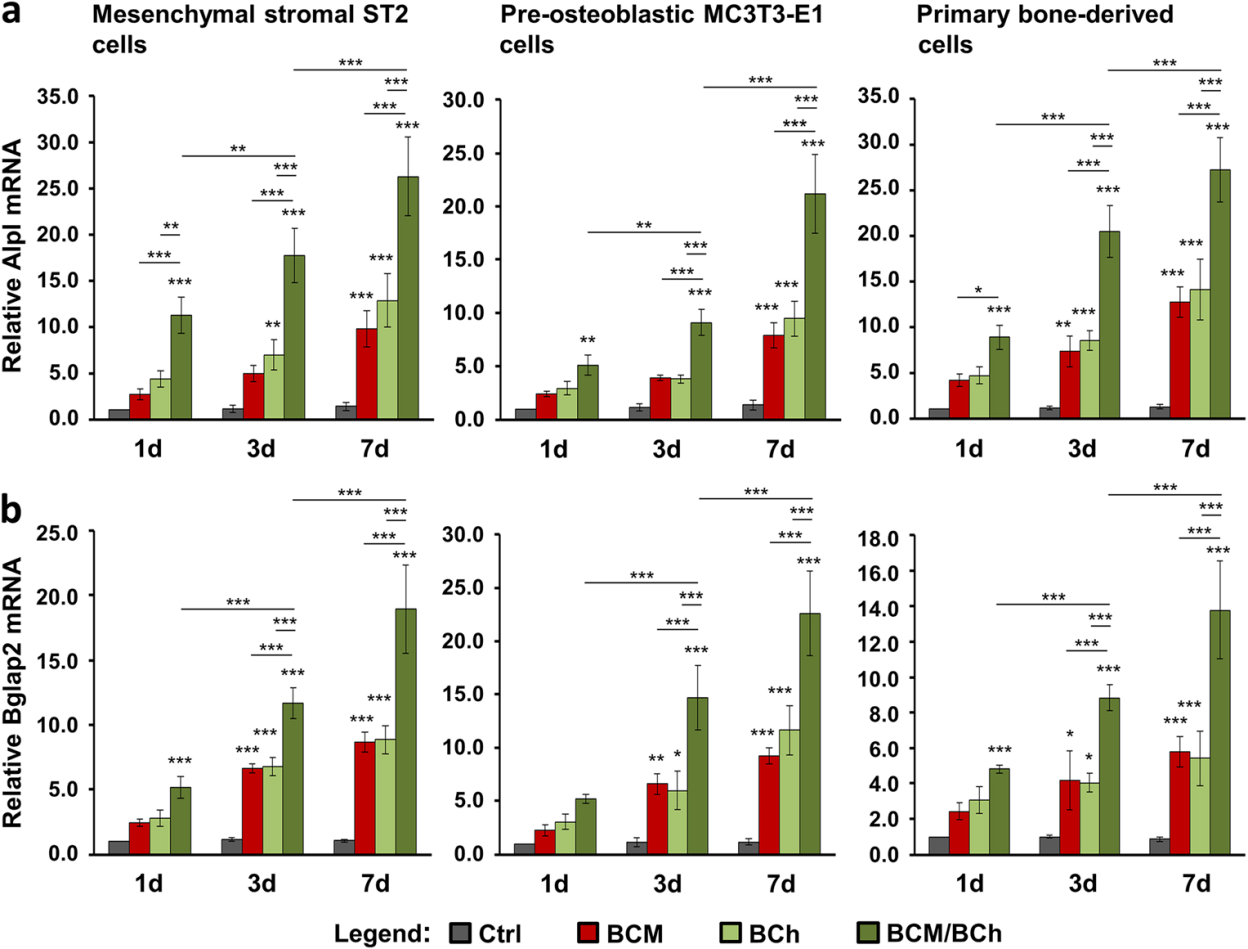
Additive effect of fresh bone chips and BCM-coated DBBM on the Alpl and Bglap2 gene expression in bone-related cell cultures. **a**, **b** Effect of BCM-coated DBBM in the absence or presence of fresh bone chips on Alpl (a) and Bglap2 (b) mRNA levels in ST2, MC3T3-E1, and primary bone–derived cells. Cells were grown under each of the four experimental conditions described in Fig. 7a for 1, 3, and 7 days before total RNA was extracted and analyzed by qRT-PCR. Values normalized to Gapdh are expressed relative to the values of control cells at the time point “1 day.” Please note the differences in the scales of the *y*-axis. Data represent means ± SD from three independent experiments. Significant differences to the respective controls at day 1 unless otherwise indicated, ***P < 0.001, **P < 0.01, *P < 0.05

The expression of the osteogenic differentiation markers Col1a1 (Fig. 7b), Runx2 (Fig. 7c), Alpl (Fig. 8a), and Bglap2 (Fig. 8b) in cells grown under the above listed conditions for 1, 3, and 7 days was analyzed by qRT-PCR. Generally, both BCM applied as a coating on DBBM (red bars) and fresh bone chips combined with BCM-free DBBM (light green bars) caused increased expression of each of the four differentation markers in all three cell types (Figs. 7 and 8). Interestingly, whereas there were no significant differences between the individual effects of BCM and fresh bone chips, there was an additive and in most of the cases extremely significant (*P* < 0.001) effect of fresh bone chips combined with BCM-coated DBBM on the osteogenic gene expression at all three time points (dark green bars). The combined effect of the fresh bone chips and BCM applied as a coating on the biomaterial appeared to be more than additive on the expression of (1) Col1a1 at day 7 in the primary bone–derived cells (Fig. 7b), (2) Runx2 at day 7 in MC3T3-E1 cells (Fig. 7c), (3) Alpl at all three time points in ST2 cells as well as at days 3 and 7 in MC3T3-E1 and primary bone–derived cells (Fig. 8a), and (4) Bglap2 at days 3 and 7 in MC3T3-E1 and the primay bone–derived cells (Fig. 8b). In contrast to Cola1a gene expression, where the cumulative increase reached a plateau at day 3 (Fig. 7b), we observed a continuous and very significant upregulation in the expression of Runx2 (Fig. 7c), Alpl (Fig. 8a), and Bglap2 (Fig. 8b) over the entire 7-day period.

In summary, the reported data demonstrate a strong additive effect of fresh bone chips combined with BCM-coated DBBM on the osteogenic differentiation of the three cell types utilized in the present study. This justifies the simultaneous application of BCM-coated DBBM and autologous bone chips leading to the complete utilization of the osteoinductive properties of the autograft.

## Discussion

DBBM is a calcium phosphate–based bone substitute of natural origin, widely used in dental surgery for bone augmentation procedures and for the treatment of periodontal and peri-implant bone defects [43–45]. By nature, DBBM is a bovine bone undergoing a chemical extraction process at low heat (300 °C) for removing existing organic components while the natural architecture of bone remains preserved [46]. Whereas some alloplastic materials exhibit considerably high elastic moduli, DBBM has an elastic modulus close to that of mandibular cortical bone [47, 48]. In vivo, it is nearly nonresorbable [49]. DBBM is one of the most researched bone grafting materials, which has demonstrated excellent osteoconductive properties positively influencing the bone regeneration [50–52]. Furthermore, it has been shown to successfully adsorb a variety of proteins, including BMPs, thus generating favorable microenvironment for migrating mesenchymal stem cells and osteoprogenitors [53, 54]. Aiming to maximize the adsorption of factors released in the BCM to a bone grafting material, we made the choice to utilize DBBM in the current in vitro study. Previous studies have demonstrated that a 24-h extraction of cortical bone chips of porcine origin with cell culture media contains a large spectrum of proteins (more than 150) [6] possessing the potential to target cellular processes in various cell types playing a role in the graft consolidation process [7–9, 32]. In particular, the BCM improved oral fibroblast cell activity [7, 8] as well as induced osteoclastogenesis in bone marrow cultures [9]. It can be suggested that BCM resembles other autogenous growth factor sources such as platelet-rich fibrin (PRF) or platelet-rich plasma (PRP). However, a number of studies have shown a limited ability of the platelet concentrates to induce bone formation [55–58]. This may be explained by the fact that the growth factors contained in PRF and PRP originate from whole blood, whereas BCM represents an autologous growth factor-rich medium originating from bone tissue and aiming to accelerate the regeneration of the same tissue. Therefore, BCM is thought to exhibit a specific activity in bone augmentation. Indeed, it has been proven that BCM triggers a prominent response of TGF-β signaling pathways by modulating the expression of adrenomedulin (ADM), pentraxin 3 (PTX3), BTB/POZ domain-containing protein 11 (BTBD11), interleukin-11 (IL-11), NADPH oxidase 4 (NOX4), and proteoglycan 4 (PRG4), all known downstream regulators of the TGF-β signaling cascade [8]. Various functions associated with bone metabolism and graft consolidation have been reported for each of the genes in this panel [8]. Moreover, our most recent research identifies TGF-β1 and BMP-2, two growth factors with a specific activity in bone regeneration, as major constituents of BCM [33]. Most importantly, a fast and significant release of TGF-β1 from autogenous bone was detected within 10 min and a specific crosstalk between TGF-β1 and BMP-2 was pointed as the mechanism by which BCM exerts its activity on the osteogenic differentiation of mesenchymal stromal cells [33].

Thus, the goal of the current study was to take a next step in investigating the biological activity of BCM by combining it with DBBM. Our data strongly suggest that bio-activation of osteoconductive bone substitutes with a short-term extracted BCM is possible. Osteogenesis is characterized by recruitment of osteoprogenitors, their attachment, proliferation, and differentiation into mature osteoblasts [59]. Indeed, bone-related cell types grown on DBBM coated with BCM, which was prepared within a clinically relevant time window of 20 min, exhibited increased adhesive, proliferative, and differentiation properties. To the best of our knowledge, this is the first study that gives a rationale for the fast preparation of BCM during the surgical implant bed preparation and its application for coating of biomaterials. Moreover, we observed an additive, nearly synergistic effect of fresh bone chips combined with BCM-coated DBBM on the osteogenic differentiation of the three cell types utilized in the study. This further supports the simultaneous application of BCM-coated DBBM and autologous bone chips in GBR procedures. In comparison to the present study where BCM was applied as a coating on DBBM, it is interesting to note that an opposing effect of BCM on the osteogenic differentiation of ST2 cells was observed with BCM applied in suspension, in the absence of a biomaterial [31, 33]. This observation was valid for both BCM preparations, 20-min and 24-h. Peng et al. detected decreased alkaline phosphatase activity and mRNA expression in ST2 cells treated with a 24-h BCM preparation [31], and we observed an inhibitory effect of the 20-min BCM preparation applied in suspension on the expression of late osteogenic markers [33]. We can thus speculate that proteins contained in the BCM may exhibit differential confirmation when coated on DBBM or another biomaterial. This new confirmation may be differentially recognized by transmembrane receptors on the cell surface leading to activation of signaling pathways different from the ones activated by proteins contained in a suspension. Altogether, our data strongly support a clinical approach combining the excellent adsorption and osteoconductive properties of the DBBM with two sources of autogenous growth factors, namely BCM and autologous bone chips. Furthermore, BCM is extracted in a mixture of Ringer’s solution and patient blood, which adds the activity of factors from yet a third autogenous source to the BCM. Indeed, BCM-RS + S preparations were more potent in inducing the osteogenic differentiation of the tested cell types. RS + S in itself was enough to stimulate cellular proliferation but not osteogenesis. The topic of blood coating of biomaterials is poorly investigated and deserves further attention. A study reports differential positive effects of titanium implant surface coating with different blood components on the proliferative potential of human osteoblasts [60]. The differences that we observed in the effects caused by the DBBM coated with the 20-min or the 24-h BCM preparation were with variable significance for the various cellular functionalities tested in the study. This may be attributed to the specific protein content and protein stability of the different BCM preparations and deserves further investigation.

A number of studies have reported combination approaches utilizing bone substitutes and recombinant growth factors. BMP-2, BMP-7, platelet-derived growth factor (PDGF), fibroblast growth factor-2 (FGF-2), and growth and differentiation factor-5 (GDF-5) are among the growth factors commonly researched and utilized in regenerative dentistry [61–67]. Terheyden et al. combined DBBM of the same type as the used in the current study with BMP-7 and compared this combination with DBBM alone in maxillary sinus floor grafting in pigs [61]. The authors reported that significant amounts of new bone were generated in both groups with the important exception that the BMP-7-coated DBBM group resulted in faster apposition and better quality of bone. Shortcomings of utilizing recombinant growth factors for clinical practice have been their short half-lives, instability and fast degradation rates, side effects, and not on a last place poor cost-effectiveness [68–71].

The approach of utilizing a combination of BCM-coated DBBM and autologous bone chips, described in the current study, would revoke the necessity of extracting a huge amount of autologous bone chips allowing an intraoral rather than an extraoral donor site to be used for autograft harvesting. This will further decrease the postoperative morbidity at the patient donor site and contribute to a reduced healing time. Combined with previous findings about the biological activity of the BCM [33], the current study represents the first steps towards the establishment of an in vitro-tested standard procedure for generation of BCM as well as for pre-coating of DBBM with it, within the limited time for the surgical tooth bed preparation.

A limitation of the present study is the use of BCM of porcine origin. Future research employing transcriptomics and proteomics analyses of RNA and protein extracted from cells grown on DBBM coated with human BCM is needed in order to determine the genes, on a genome-wide base, whose expression have been modulated in response to short-term extracted human BCM. These additional investigations would supplement the data presented in the current study by investigating the signaling pathways of osteogenesis triggered by the BCM-activated biomaterial. Furthermore, recommendation stemming from in vitro studies in the form of a protocol for the preparation of BCM and its application for biofunctionalization of DBBM in GBR procedures would need an animal testing in vivo. Such studies will determine whether BCM combined with a bone grafting material may be a superior candidate for bone regeneration procedures than the individual recombinant growth factors combined with the respective bone graft.

In summary, the presented in vitro study clearly demonstrates that biological pre-activation of DBBM with BCM, extracted within a clinically relevant time window of 20 min, is feasible and may appear as an optimal modality in the treatment of both regular and complex bone defects.

## Electronic Supplementary Material

ESM 1 (PDF 665 kb)

## Data Availability

All data generated and analyzed during this study are included in this article and its Electronic Supplementary Material file.
