# Supplementary material for: Cellular responses to deproteinized bovine bone mineral biofunctionalized with bone-conditioned medium
Source: Clin Oral Investig. 2020 Sep 1;25(4):2159–73. doi: 10.1007/s00784-020-03528-6 (PMC7966141; doi:10.1007/s00784-020-03528-6)
Supplement: Supplementary file 1 — (PDF 665 kb) [file 784_2020_3528_MOESM1_ESM.pdf]

Electronic Supplementary Material for:

Cellular responses to deproteinized bovine bone mineral biofunctionalized with bone-conditioned medium (BCM)

Ludovica Parisi[1,2,a,b], Daniel Buser[2], Vivianne Chappuis[2,c] and Maria B. Asparuhova[1,2,c*]

[1]Laboratory of Oral Cell Biology, Dental Research Center, School of Dental Medicine, University of Bern, Freiburgstrasse 3, 3010 Bern, Switzerland

[2]Department of Oral Surgery and Stomatology, School of Dental Medicine, University of Bern, Freiburgstrasse 7, 3010 Bern, Switzerland

[a]Permanent address: Centro Universitario di Odontoiatria, Dipartimento di Medicina e Chirurgia, University of Parma, Via Gramsci 14, 43126 Parma, Italy

[b]Present address: Laboratory of Oral Molecular Biology, Dental Research Center, Department of Orthodontics and Dentofacial Orthopedics, University of Bern, Freiburgstrasse 3, 3010 Bern, Switzerland

[c]These authors share senior authorship

*Correspondence: mariya.asparuhova@zmk.unibe.ch; Tel.: +41 31 632 86 22

Content:

Figure S1. Characterization of primary human bone-derived cells.

Figure S2. Supplementary information for cell adhesion assay.

Table S1. Primer sequences for mouse genes.

Table S2. Primer sequences for human genes.

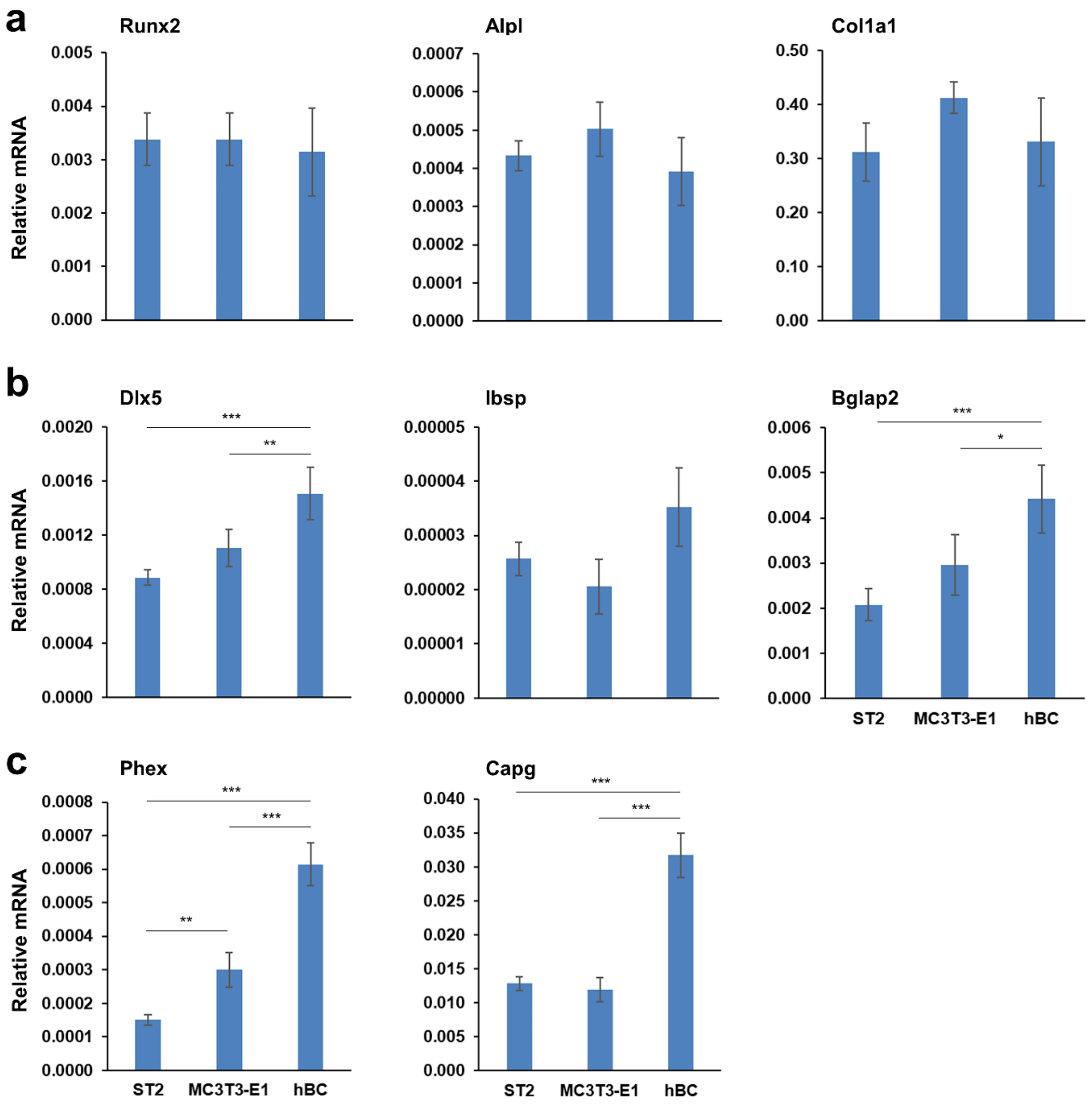

Figure S1. Characterization of primary human bone-derived cells. Primary human bone-derived cells (hBC) were characterized, in comparison with mesenchymal stromal ST2 and pre-osteoblastic MC3T3-E1 cells, for the expression of (**a**) osteoprogenitor (Runx2) and pre-osteoblast (Alpl and Col1a1) markers, (**b**) osteoblast markers (Dlx5, encoding the distal-less homeobox 5; Ibsp, encoding the integrin-binding sialoprotein; and Bglap2), and (**c**) osteocyte markers (Phex, encoding the phosphate regulating endopeptidase homolog, X-linked; Capg, encoding the capping actin protein, gelsolin like; and Sost, encoding sclerostin). ST2, MC3T3-E1, and hBC cells were starved in 0.3% FCS-containing medium for 24 h before total RNA was extracted and analyzed for the expression of the above-mentioned transcripts by qRT-PCR. Experimental values were normalized to Gapdh and analyzed by the ΔCt method. Data

represent means ± SD from three independent experiments. Significant differences ***P < 0.001, **P < 0.01, and *P < 0.05 are shown.

In contrast to the osteoprogenitor ST2 and pre-osteoblastic MC3T3-E1 cells, the primary hBCs were characterized as a mixture of cells possessing osteoblastic and preosteocytic phenotype. No differences in the expression of early osteogenic differentiation markers were detected between the three cell types whereas most of the later osteogenic markers tested were significantly upregulated in the primary hBCs. However, no expression of sclerostin, which is considered as a marker of mature osteocytes, was detected in any of the three cell types.

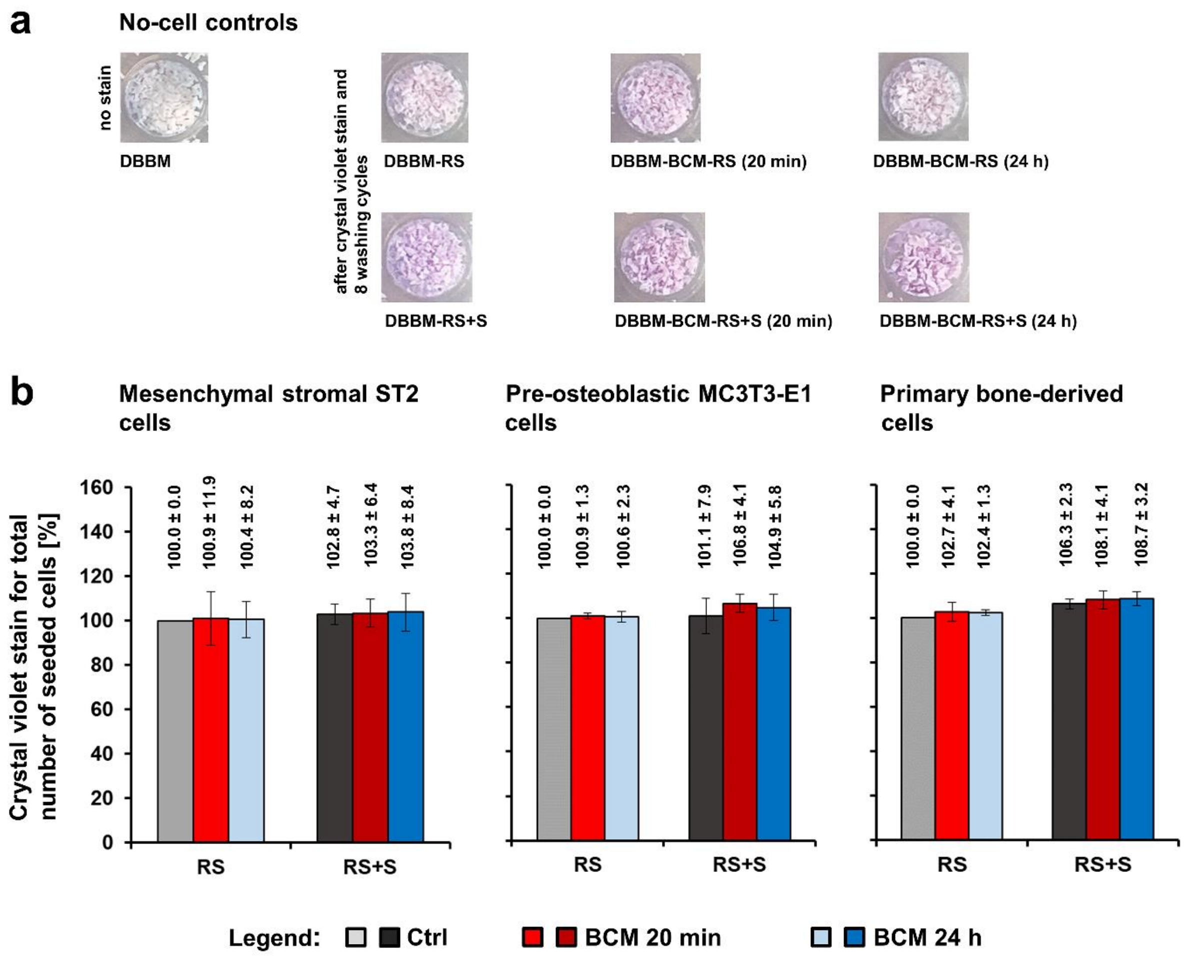

Figure S2. Supplementary information for cell adhesion assay. (a) Representative images of no-cell controls. An image of DBBM sample without crystal violet staining is shown on the left. The remaining images, shown in the middle and right, are taken after crystal violet staining in 0.1% solution made in PBS and 8 washing cycles in deionized H_2O. 35 mg of DBBM granules were placed on the bottom of 96-well ultra-low attachment plates and pre-hydrated with Ringer's solution (DBBM-RS) or pre-coated with either Ringer's solution mixed with autogenous serum (DBBM-RS+S) or each of the BCMs extracted within 20 min or 24 h in each of the two diluents (RS or RS+S). The images demonstrated a slight background generated by both DBBM and BCM. **(b)** Quantitative assessment of background stain produced by BCM in a crystal violet cell adhesion assay. 5 x 10^4 cells/well of ST2, MC3T3-E1, or primary bone-derived cells were plated on DBBM coated with BCM-RS or BCM-RS+S

prepared within 20 min or 24 h each. Controls (Ctrl) represent cells of each cell type seeded on BCM-free DBBM hydrated with RS or RS+S. After 1 h of incubation, the culture medium was removed and the cells were directly fixed in 4% PFA, omitting the 3 cycles of extensive wash in PBS, followed by a crystal violet staining. After 8 washing cycles in deionized H_2O to remove crystal violet excess, the dye bound to the cells was solubilized using a 10% acetic acid on an orbital shaker at 150 rpm for 5 min at room temperature. The absorbance of the eluate was measured at 570 nm using an EL808 reader. That way, a signal for the total number of cells seeded on an equal amount of DBBM in each experimental group was obtained. A comparison to the signal, produced by the total number of control cells seeded on DBBM hydrated with RS (light grey bars), taken as 100%, revealed average background levels of 1) 3.4% produced by RS+S, 2) 1.3% produced by BCM made in RS, and 3) 5.9% produced by BCM made in RS+S. Means ± SD from three independent experiments are shown.

Table S1. Primer sequences for mouse genes.

Gene symbol	Gene bank accession number	Primer pair (fwd/rev)	Amplicon size (bp)
Col1a1	NM_007742.3	5′- CCGGAAGAATACGTATCACCA-3'<br>5′- TCTGGGAAGCAAAGTTTCCT-3'	199
Runx2	NM_001271627.1	5′- AGGGACTATGGCGTCAAACA-3'<br>5′- GGCTCACGTCGCTCATCTT-3'	137
Alpl	NM_001287172.1	5′- GCAACTCCATCTTTGGTCTG-3'<br>5′- GTTGTTGTGAGCGTAATCTACC-3'	145
Bglap2	NM_001032298.3	5′- CACCTAGCAGACACCATGAG-3'<br>5′- TGGACATGAAGGCTTTGTCAG-3'	123
Gapdh	NM_008084.3	5′-CTTGTGCAGTGCCAGCCTC-3'<br>5′-GCCGTGAGTGGAGTCATACTG-3'	189
Dlx5*	NM_198854	5′- GTCCCAAGCATCCGATCCG-3'<br>5′- GCGATTCCTGAGACGGGTG-3'	79
Ibsp*	NM_008318.3	5′- GGTCTTTAAGTACCGGCCAC-3'<br>5′- CGTTTGAAGTCTCCTCTTCCTC-3'	157
Phex*	NM_011077	5′- GAAAGGGGACCAACCGAGG-3'<br>5′- AACTTAGGAGACCTTGACTCACT-3'	105
Capg*	NM_007599	5′- ACACACCCATCCCTCAGAGT-3'<br>5′- CTGGCCTATCCACAGGTGC-3'	197

*Genes used in the supplementary Figure S1.

Table S2. Primer sequences for human genes.

Gene symbol	Gene bank accession number	Primer pair (fwd/rev)	Amplicon size (bp)
COL1A1	NM_000088.4	5′- GAAGGGACACAGAGGTTTCAG-3'<br>5′- TAGCACCATCATTTCCACGA-3'	190
RUNX2	NM_001015051.3	5′- AGACCAACAGAGTCATTTAAGGC-3'<br>5′- GGTGTCACTGTGCTGAAGAG-3'	115
ALPL	NM_000478.6	5′- TGGCAACTCTATCTTTGGTCTG-3'<br>5′- TTGTTGTGAGCATAGTCCACC-3'	146
BGLAP	NM_199173.6	5′- GTGCAGAGTCCAGCAAAGGT-3'<br>5′- TCAGCCAACTCGTCACAGTC-3'	175
GAPDH	NM_001256799.3	5′- ATCAAGAAGGTGGTGAAGCAG-3'<br>5′- TCGTTGTCATACCAGGAAATGAG-3'	178
DLX5*	NM_005221	5′- TTCCAAGCTCCGTTCCAGAC-3'<br>5′- GAATCGGTAGCTGAAGACTCG-3'	86
IBSP*	NM_004967.3	5′- GGAATGGCCTGTGCTTTCTC-3'<br>5′- AGTCACTACTGCCCTGAACTG-3'	162
PHEX*	NM_000444	5′- TTTCTTCCGGTTCGCTTGTGA-3'<br>5′- AGTTCCTTCAACTTGAGGTCAAC-3'	117
CAPG*	NM_001256140	5′- AGTCAGCATTTCACAAGACCTC-3'<br>5′- CACCACACCAGGCGAAGAT-3'	174

*Genes used in the supplementary Figure S1.
